# Validación de la metodología para cuantificar el fluconazol y sus impurezas orgánicas en materia prima por cromatografía líquida de alta resolución

**DOI:** 10.7705/biomedica.6850

**Published:** 2023-08-31

**Authors:** James Alexander Castillo, Natalia Afanasjeva

**Affiliations:** 1 Programa de Química, Universidad del Valle, Cali, Colombia Universidad del Valle Programa de Química Universidad del Valle Cali Colombia; 2 Departamento de Química, Facultad de Ciencias Naturales y Exactas, Universidad del Valle, Cali, Colombia Universidad del Valle Departamento de Química Facultad de Ciencias Naturales y Exactas Universidad del Valle Cali Colombia

**Keywords:** estudios de evaluación como asunto, cromatografía líquida, estudio de validación, fluconazol, contaminación de medicamentos, química analítica, Evaluation studies as topic, chromatography, liquid, validation study, fluconazole, drug contamination, chemistry, analytic

## Abstract

**Introducción.:**

La eficiencia de una metodología para analizar una sustancia farmacológica puede verse afectada por las condiciones reales del laboratorio de cada país, incluyendo el clima. Por esta razón, se requiere validar el método con las pautas recomendadas para ello y optimizar el proceso, para asegurar el éxito y la confianza en los resultados.

**Objetivo.:**

Validar una metodología para la cuantificación simultánea del fluconazol (materia prima) y sus impurezas orgánicas mediante cromatografía líquida de alta resolución con detector de arreglo de diodos en condiciones de clima tropical y con todos los requisitos normativos.

**Materiales y métodos.:**

Se hicieron pruebas previas a la validación del método: idoneidad del sistema, estudio de filtros, límite de cuantificación, ausencia del error sistemático, estudios de degradación forzada y estabilidad de las soluciones. Además, se validaron: la especificidad, la linealidad, la exactitud, la precisión y la robustez.

**Resultados.:**

La pureza espectral del método se logró al obtener la separación de los productos de degradación de los picos de los analitos. La estabilidad de las soluciones no se vio afectada, en la frecuencia evaluada de 24 horas, a temperatura ambiente y de refrigeración. Se obtuvo una linealidad con coeficientes de correlación mayores o iguales a 0,999 para la valoración y mayores o iguales a 0,997 para las impurezas. La recuperación estuvo en el rango de 98 a 102,0 % de fluconazol, con una exactitud entre el 80 y el 120 % para las impurezas. El factor de repetibilidad y reproducibilidad no superó la desviación estándar relativa del 2,0 % para la valoración y, la del 5,0 %, para las impurezas, lo cual mostró una solidez adecuada del método. Además, se obtuvo un tiempo corto de ejecución del análisis, lo que permitió la rápida determinación de la calidad de la materia prima.

**Conclusión.:**

Se demostró que el método de cuantificación de fluconazol, validado por cromatografía líquida de alta resolución con detector de arreglo de diodos, es lo suficientemente selectivo, preciso, exacto, lineal y robusto; además, es capaz de generar resultados analíticos veraces en condiciones de uso reales, incluyendo el clima tropical de Colombia.

## Abstract (en)

**Introduction.:**

The real laboratory conditions of each country, including climate, can affect the method's efficiency in analyzing a pharmacological substance. Thus, it is necessary to validate the process according to the corresponding guidelines and optimize it to ensure success and confidence in the results.

**Objective.:**

The objective was to validate a methodology for fluconazole and its organic impurities quantification in raw material using high-performance liquid chromatography, with a diode array detector, under tropical climate conditions, and complying with all regulatory requirements.

**Materials and methods.:**

We performed pre-validation tests of the method consisting of system adequacy, filters study, quantification limit, absence of systematic error, forced degradation studies, and solutions stability. In addition, we validated the specificity, linearity, accuracy, precision, and robustness of the system.

**Results.:**

Separation of the degradation products from the analyte peaks allowed the achievement of the method's spectral purity. The solution's stability was not affected during the evaluated time (24 hours) at room temperature and under refrigeration. Linearity resulted in correlation coefficients greater than or equal to 0.999 for the evaluation and greater than or equal to 0.997 for impurities. We obtained a fluconazole recovery varying from 98 to 102% with an accuracy between 80 to 120% for impurities detection. The repeatability and reproducibility factor did not exceed a relative standard deviation of 2.0% for the evaluation and of 5.0% for the impurities, demonstrating the adequate robustness of the method. In addition, a short analysis execution time allowed the quick determination of the raw material quality.

**Conclusion.:**

We demonstrated that the fluconazole quantification method validated by high-performance liquid chromatography is sufficiently selective, precise, exact, linear, and robust to generate accurate analytical results under real conditions, including the tropical climate of Colombia.

Los medicamentos son sustancias elementales para mejorar la calidad de vida de las personas, por lo que su consumo, dosificación, administración y proceso de fabricación deben ser rigurosamente controlados. Este último requiere de la vigilancia y la supervisión constante, y de un proceso de calidad óptimo desde la adquisición de las materias primas hasta el embalaje del producto final, que cumpla con las pautas establecidas para garantizar la confiabilidad y la seguridad del fármaco para su consumo. La eficiencia de los resultados del método analítico puede afectarse por las condiciones reales de análisis de cada laboratorio farmacológico en un país o una zona climática determinada. En consecuencia, es necesario validar la metodología con las condiciones reales de análisis, para optimizar el proceso y obtener resultados confiables.

En este trabajo se desarrolló el método de análisis de la materia prima fluconazol, conocido químicamente como (2-(2,4-difluorofenil)-1,3-bis(1,2,4-triazol-1-il)propan-2-ol) ^1^. Es un ingrediente farmacéutico activo, un antifúngico clasificado en el grupo de los azoles, más específicamente, como un triazol ^2^. Se administra para tratar infecciones producidas por hongos ^3^^,^^4^ Es ampliamente usado para tratar casos de infecciones como la candidiasis de las mucosas e infecciones sistémicas, incluidas la candidiasis, la coccidioidomicosis y la criptococosis ^5^^,^^6^. Actúa inhibiendo la desmetilación del citocromo P-450 fúngico (C-14-α-desmetilasa), lo que resulta en la acumulación de 14- α -metil-esteroles fúngicos, la pérdida de los esteroles fúngicos normales y la actividad fungistática. Estos efectos interfieren con la adhesión celular y la biosíntesis del ergosterol, lo cual altera la membrana y el crecimiento de la pared en las células fúngicas ^1^.

Una de las técnicas actuales más eficientes para obtener resultados óptimos y confiables, es el análisis por cromatografía líquida de alta resolución *(High Performance Liquid Chromatography,* HPLC), con absorción UV-visible, mediante el uso de espectrómetros de arreglos de diodos (DAD) ^7^^-^^10^. Es una herramienta fundamental para evaluar diversos fármacos y sus validaciones ^11^^,^^12^. Por esto, se desarrolló y validó una metodología para la valoración del ingrediente farmacéutico activo del fluconazol (materia prima) y la cuantificación simultánea de sus impurezas orgánicas por HPLC-DAD en fase inversa.

En esta evaluación, se tuvieron en cuenta las condiciones del clima tropical de Colombia, de acuerdo con las pautas establecidas en la monografía de la farmacopea de los Estados Unidos ^13^, y el ajuste de los parámetros, según lo permitido por la Conferencia Internacional de Armonización ^14^. Esto, con el fin de optimizar el proceso de análisis, y la reducción del tiempo y los costos del proceso, ya que, al fijar unos parámetros específicos, se puede garantizar la eficiencia del análisis y el cumplimiento de sus regulaciones.

En este trabajo, se analizaron muestras del ingrediente farmacéutico activo fluconazol y los resultados de la validación del método se utilizaron para estimar la calidad, la confiabilidad y la reproducibilidad del análisis, como un aspecto fundamental de las buenas prácticas de laboratorio ^15^.

## Materiales y métodos

### Equipos, estándares y reactivos

Se usó un equipo de cromatografía líquida Vanquish™ Core HPLC (Thermo Scientific), equipado con una bomba cuaternaria C, un automuestreador Split Sampler CT, un detector con arreglo de diodos y un compartimiento de columna C Vanquish™ equipado con un *software* para la recopilación y el procesamiento de los datos cromatográficos, denominado Thermo Scientific Dionex Chromeleon™ 7 Chromatography Data System, versión 7,3. Se utilizó una columna cromatográfica empaquetada de tipo C18 Phenomenex Prodigy™ de 3 µm ODS-3, con un diámetro de partícula de 3 µm, un tamaño de poro de 100 Å, un diámetro interno de 4,6 mm y una longitud de 150 mm. Para la reproducibilidad, se empleó un equipo de cromatografía líquida QSM-R Waters™ Acquity Arc con una bomba cuaternaria, un automuestrador Sample Manager y un detector con arreglo de fotodiodos 2998 con el *software* Empower® 3 Pro Chromatography Data System, versión 7,4. El tratamiento estadístico de los datos y la interpretación de los resultados se llevaron a cabo mediante el *software* MVA™ (NOVIA Gmb), versión 2,1.

La fase móvil se filtró con un equipo de filtración al vacío, usando una presión de 0,035 MPa y una membrana hidrofílica de polifluoruro de vinilideno (PVDF) (QLS™) con un tamaño de poro de 0,45 µm. Además, se usaron los siguientes equipos: un baño ultrasónico Cole-Parmer 8894R-DTA, una balanza analítica Sartorius Secura® 225D-1S, unas micropipetas Brand Transferpette® S D-100 (10 µl-1000 µl) y D-1000 (100 µl-1000 µl), una estufa universal Memmert UNB 500, un baño de agua Memmert WNB 14, una cabina con intensidad lumínica entre 2000 lx y 3750 lx (según el Luxómetro Testo 540) y una nevera vertical Refrimag con temperatura de 2 a 8 °C. Se emplearon filtros para jeringa con membrana hidrofílica PVDF de 0,45 µm (QLS™) y material volumétrico en vidrio clase A de 10,0 ml, 20,0 ml, 25,0 ml y 50,0 ml, marca Duran™. Los estándares, los reactivos y las muestras utilizadas en esta validación, se muestran en el cuadro 1.

**Cuadro 1:** Estándares, reactivos y muestra utilizados en la validación del fluconazol

Nombre	Grado	Fabricante	Lote	Potencia (%)
Fluconazol Supelco®	Secundario	Sigma-Aldrich	LRAC0255	99,96
Fluconazol - compuesto relacionado A	Primario	USP	R05480	100,00
Fluconazol - compuesto relacionado B	Primario	USP	R05370	97,00
Fluconazol - compuesto relacionado C	Primario	USP	R061Y0	99,90
Acetonitrilo Chromasolv™	HPLC	Honeywell	EC316-CO	99,98
Agua	HPLC	Laboratorio	N/A	N/A
Peróxido de hidrógeno Perdrogen™ 30 % (w/w)	RA	Honeywell	J2000	30,10
Ácido clorhídrico 37 % ExpertQ®	RA	Scharlau	21344209	37,98
Hidróxido de sodio, pellets Emprove® Essential	RA	Merck®	B1669382	99,20
Fluconazol	MP	Virupaksha Organics Limited, India	AFLNC0221027(M)	100,30

HPLC: *High Performancee Liquid Chromatography,* USP: *United States Pharmacopeia,* máximo grado de pureza; N/A: no aplica; RA: reactivo analítico; MP: materia prima

### Pautas base para la validación de la metodología analítica

La validación de la metodología se propuso según las pautas establecidas por la Conferencia Internacional de Armonización ^14^, la guía para la industria de validación de métodos bioanalíticos de la *Food and Drug Administration* de Estados Unidos ^16^ y el capítulo sobre "Validación de procedimientos compendiales", teniendo en cuenta los parámetros de desempeño de la categoría I para la valoración, y los de la categoría II para las impurezas ^17^. Para la valoración y la identificación de impurezas orgánicas del fluconazol (materia prima) por HPLC-DAD, se tomó como base la metodología analítica descrita en la farmacopea de Estados Unidos ^13^.

### Condiciones cromatográficas del método

La técnica cromatográfica se validó mediante un sistema en fase inversa y una mezcla isocrática del reactivo acetonitrilo y de agua, de grado HPLC, en una proporción de 20:80 (v/v); con una temperatura de columna de 40 °C, una longitud de onda de 260 nm, un volumen de inyección de 20 µl con flujo de 0,5 ml/minuto y un detector con arreglo de diodos.

### Preparación de la solución blanco, el estándar de impurezas, el estándar de fluconazol y la solución muestra para la valoración y la detección de impurezas

Se usó la fase móvil como diluyente y blanco. Se empleó un filtro para jeringa con membrana hidrofílica de PVDF con poro de 0,45 µm y se transfirió a un vial incoloro para HPLC. Para la detección de impurezas, se prepararon soluciones madre de 0,5 mg/ml de cada estándar de referencia (cuadro 1) de fluconazol, fluconazol compuesto relacionado A, fluconazol compuesto relacionado B y fluconazol compuesto relacionado C. Estos se disolvieron en acetonitrilo y se llevaron al volumen deseado con la fase móvil filtrada. A partir de estas soluciones madre, se procedió a preparar la solución estándar mixta de impurezas con una concentración final de 10 µg/ml. Para la valoración, se preparó el estándar, a partir del fluconazol de referencia, a una concentración final de 0,5 mg/ml. Las muestras se prepararon con el fluconazol materia prima a una concentración final de 0,5 mg/ml para la valoración y una de 3,0 mg/ml para las impurezas. Todas las soluciones se llevaron a un volumen final de 50 ml con la fase móvil.

### Pruebas de prevalidación (idoneidad del sistema, prueba de filtros, determinación de la estabilidad de las soluciones)

**Figura 1:**
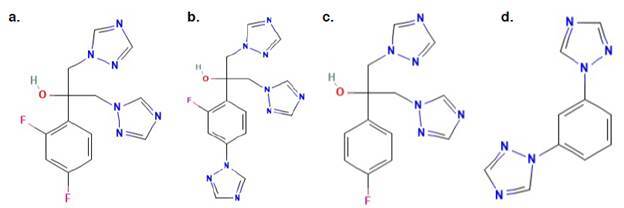
(a) fluconazol (2-(2,4-difluorofenil)-1,3-bis(1,2,4-triazol-1-il)propan-2-ol) (1); (b) fluconazol - compuesto relacionado A (2-[2-fluoro-4-(1H-1,2,4-triazol-1-il)fenil]-1,3-bis(1H-1,2,4-triazol-1-il)-propan-2-ol) ^18^; (c) fluconazol - compuesto relacionado B (2-(4-fluorofenil)-1,3-bis(1H-1,2,4-triazol-1-il)propan-2-ol) ^19^; (d) fluconazol - compuesto relacionado C (1,3-Di(1H-1,2,4-triazol-1-il)benceno) ^20^

Se hicieron pruebas al sistema -instrumentos, analistas, equipos y sustancias de referencia- para verificar su operación con los criterios preestablecidos, que permitieron garantizar la confiabilidad de los resultados de la metodología analítica propuesta ^13^. En la solución estándar mixta de impurezas, se verificó la correcta elución cromatográfica (cuadro 2) de cada pico de impurezas en el fluconazol. Las estructuras químicas de los compuestos evaluados se presentan en la figura 1.

**Cuadro 2:** Tiempos de retención relativa, con respectivo orden cromatográfico de elución (13)

Nombre	Tiempo de retención relativa (minutos)
Fluconazol - compuesto relacionado A	0,50
Impureza especifica	0,60
Fluconazol - compuesto relacionado B	0,81
Fluconazol - compuesto relacionado C	0,86
Fluconazol	1,00

En la prueba de filtros, la solución estándar de fluconazol, usada para la valoración, se filtró bajo dos condiciones: en la primera, se descartaron los primeros 0,5 ml y, en la segunda, se descartó el primer mililitro. El volumen descartado se transfirió a tres viales independientes, según la condición.

La estabilidad de las soluciones se determinó a partir de la preparación de la solución estándar de impurezas (estándar 1), el estándar 2 de valoración y un conjunto de tres muestras para la valoración. Este análisis se registró como tiempo cero. Se hicieron alícuotas por separado de las soluciones y las muestras, y se almacenaron a temperatura ambiente o se refrigeraron entre 2 y 8 °C. La estabilidad de las soluciones se evaluó nuevamente para las dos condiciones, en un periodo de 24 horas.

### Límite de cuantificación

La prueba de límite de cuantificación, en la que cada analito puede cuantificarse confiablemente ^21^, se practicó a partir de los resultados previos de la solución estándar de impurezas con concentración de 10 µg/ml y se extrapoló la proporción señal/ruido a valores cercanos a 25. De esta manera, la solución para el límite de cuantificación se preparó a partir de las soluciones madre del estándar de impurezas con una concentración de 0,5 mg/ml.

Se tomaron alícuotas de 10 µl para preparar el fluconazol compuesto relacionado A; de 220,0 µl para el fluconazol compuesto relacionado B; de 12 µl para el fluconazol compuesto relacionado C, y de 234 µl para el fluconazol ingrediente farmacéutico activo (materia prima). Los compuestos se disolvieron y se llevaron a un volumen final de 20 ml con fase móvil. La concentración final obtenida fue: de 0,25 µg/ml para el fluconazol compuesto relacionado A; de 5,50 µg/ml para el fluconazol compuesto relacionado B; de 0,30 µg/ml para el fluconazol compuesto relacionado C, y de 5,85 µg/ml para el fluconazol ingrediente farmacéutico activo.

### Estudios de degradación forzada en las muestras para determinar su especificidad

Para determinar la especificidad de los compuestos, se preparó una solución blanco, una control, dos de hidrólisis ácida (1,0 N y 0,1 N), dos de hidrólisis básica (1,0 N y 0,1 N), una de oxidación, una de termólisis y una solución sometida a fotólisis forzada. Las soluciones se sometieron a diferentes condiciones de degradación con calentamiento en baño de agua a 60 °C, cada una con un tiempo de corrido cromatográfico de 60 minutos.

*Preparación de la solución blanco y de la solución control.* En un balón volumétrico de 50 ml, que contenía 25 ml de diluyente, se adicionó 1,0 ml de 1,0 N de ácido clorhídrico, 1,0 ml de 1,0 N de hidróxido de sodio y 1,0 ml de peróxido de hidrógeno al 0,3 %. La solución se calentó por 30 minutos, se dejó enfriar a temperatura ambiente y se llevó a volumen con fase móvil. La solución de control se preparó como se indica para la solución muestra de impurezas, a una concentración final de 3,0 mg/ml, sin someterse a ninguna degradación.

### Condiciones de preparación de la solución muestra sometida a degradación

*Hidrólisis ácida.* En un balón volumétrico de 50 ml, se pesaron 150 mg de muestra de fluconazol, se agregaron 25 ml de diluyente y los compuestos se mezclaron hasta su completa disolución. Luego, se adicionó 1 ml de 1,0 N de ácido clorhídrico, se calentó por 30 minutos, se dejó enfriar a temperatura ambiente y se neutralizó con 1,0 ml de 1,0 N de hidróxido de sodio.

*Hidrólisis básica.* En un balón volumétrico de 50 ml, se pesaron 150 mg de muestra de fluconazol, se agregaron 25 ml de diluyente y los compuestos se mezclaron hasta su completa disolución. Luego, se adicionó 1,0 ml de 1,0 N de hidróxido de sodio, se calentó por 30 minutos, se dejó enfriar a temperatura ambiente y se neutralizó con 1,0 ml de 1,0 N de ácido clorhídrico.

*Oxidación.* En un balón volumétrico de 50 ml, se pesaron 150 mg de muestra de fluconazol, se agregaron 25 ml de diluyente y los compuestos se mezclaron hasta su completa disolución. Luego, se adicionó 1,0 ml de 0,3 % de peróxido de hidrógeno, se calentó por 30 minutos y se dejó enfriar.

*Termólisis.* En un vaso de precipitado, se pesaron 150 mg de muestra de fluconazol y se sometieron a una temperatura de 100 °C en un horno durante 24 horas. Se dejó enfriar y luego se agregaron 20 ml de diluyente. Los compuestos se mezclaron hasta su completa disolución y la mezcla se transfirió a un balón volumétrico de 50,0 ml.

*Fotólisis.* En un balón volumétrico de 50 ml, se pesaron 150 mg de muestra de fluconazol, que se expusieron durante 24 horas a una luz directa en cabina con una intensidad lumínica entre 2000 y 3750 lx. Luego, se agregaron 25 ml de diluyente y se mezclaron hasta su completa disolución.

Todas estas soluciones se llevaron a volumen con fase móvil.

### Linealidad del sistema para valorar la materia prima y detectar impurezas orgánicas del fluconazol

Se preparó la linealidad del sistema de fluconazol para su valoración a 0,50 mg/ml (100 %). Se hicieron diluciones por triplicado con concentraciones finales de 0,45 mg/ml (90 %), 0,48 mg/ml (96 %), 0,50 mg/ml (100 %), 0,53 mg/ml (106 %) y 0,55 mg/ml (110 %). De igual modo, para detectar las impurezas de fluconazol (10 µg/ml) en los compuestos relacionados A, B y C, y el fluconazol de grado ingrediente farmacéutico activo, se preparó la linealidad del sistema de impurezas orgánicas, haciendo diluciones por triplicado para obtener un límite de cuantificación de 90 %, 100 %, 110 % y 120 %. La concentración usada del compuesto relacionado A fue 0,25 µg/ml, la del compuesto relacionado B fue 5,50 µg/ml, y la del compuesto relacionado C fue 0,30 µg/ml; las diluciones de la materia prima de fluconazol quedaron a concentraciones finales de 5,85 µg/ml, 9 µg/ml, 10 µg/ml, 11 µg/ml y 12 µg/ml.

### Exactitud para valorar y detectar las impurezas orgánicas de fluconazol

Este parámetro se evaluó en el rango de la linealidad. Se analizaron tres puntos (90 %, 100 % y 110 %) para la valoración, y se seleccionaron como limité de cuantificación 100 % y 120 % para las impurezas, a partir de la concentración nominal del método para la valoración del analito y la cuantificación de las impurezas orgánicas.

### Precisión para valoración e impurezas orgánicas de fluconazol

*Repetibilidad del sistema de fluconazol.* Se determinó a partir de seis inyecciones de la solución estándar de valoración, con la concentración analítica de trabajo del fluconazol que es 0,5 mg/ml; y seis inyecciones de la solución estándar de impurezas con la concentración analítica de trabajo del compuesto relacionado A, B y C, y el fluconazol materia prima de 10 µg/ml.

*Reproducibilidad del método y precisión intermedia de fluconazol.* Para su determinación, el analista 1 y el analista 2 prepararon dos series (serie 1 y serie 2) de seis muestras, cada una con la concentración analítica de trabajo para la valoración de fluconazol (0,5 mg/ml) y para la detección de impurezas orgánicas a la concentración analítica de trabajo de 10 µg/ml para los compuestos relacionados A, B y C, y fluconazol materia prima.

*Parámetro de solidez (robustness).* Este parámetro se estimó a partir de las siguientes variaciones: en días diferentes de análisis (día 1 y 2), en equipos diferentes (equipo 1 y 2) y por analistas diferentes (analista 1 y 2), tanto para la valoración como para la detección de impurezas orgánicas del fluconazol.

## Resultados

### Idoneidad del sistema

En la valoración se encontró que, para el estándar 1 de fluconazol había una simetría menor o igual a 2, con una desviación estándar relativa menor o igual a 2 % (para seis inyecciones consecutivas), mientras que, para el estándar 2 de valoración, se halló una correlación entre 98 y 102 %.

En el estándar de impurezas orgánicas se encontró, para todos los picos, una desviación estándar relativa menor o igual a 5 % y una resolución mayor o igual a 1,5 entre el fluconazol y los compuestos relacionados B y C. La desviación estándar relativa calculada para las respuestas de las seis inyecciones de límite de cuantificación fue menor o igual al 10 %. Se calculó una recomendada entre señal y ruido de 20 a 30, considerando la variabilidad de los equipos, aunque una relación entre señal y ruido de 10:1 era aceptable para estimar el límite de cuantificación, de acuerdo con las pautas de cromatografía señaladas por la farmacopea de Estados Unidos ^21^.

### Estandarización de la metodología

*Prueba de filtros.* El porcentaje de recuperación para cada muestra estuvo en el rango de 98 a 102%. Las muestras no presentaron señales aportadas por los filtros que pudieran interferir en la identificación o cuantificación del analito de interés. La desviación estándar relativa de las respuestas de tres réplicas fue menor o igual a 2 %; y para las respuestas entre las seis inyecciones repetidas de la solución estándar sin filtrar fue menor o igual a 1 %.

**Figura 2:**
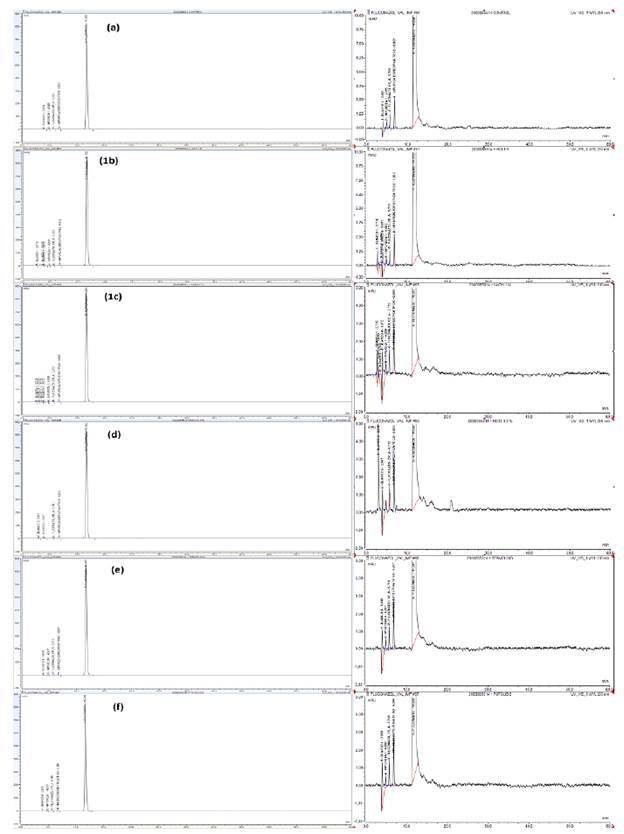
Las imágenes del lado izquierdo muestran los cromatogramas de fluconazol degradado por diferentes procesos: **(a)** muestra control de fluconazol (ingrediente farmacéutico activo), **(b)** hidrólisis ácida con 1,0 N de ácido clorhídrico, **(c)** hidrólisis básica con 1,0 N de hidróxido de sodio, **(d)** oxidación, **(e)** termólisis, **(f)** fotólisis. En el lado derecho se observan los mismos cromatogramas, pero a escala ampliada.

*Degradaciones forzadas para fluconazol.* En las pruebas de degradación forzada se observó que el fluconazol presentó una alta estabilidad. Se verificó la ausencia de interferencias de los posibles productos de degradación en el método analítico con respecto al tiempo de retención del fluconazol (figura 2, a-f). Las condiciones de estrés no afectaron la estabilidad del fluconazol dado que los porcentajes de degradación fueron muy bajos.

*Estabilidad de soluciones.* La correlación entre el estándar 1 y el estándar 2 estuvo entre el 99 y el 101 % para las inyecciones iniciales de chequeo. Para las inyecciones intermedias, y al final de la secuencia, la correlación fue del 98 al 102 % con la solución estándar de valoración. La recuperación calculada entre el contenido promedio a las 24 horas respecto al obtenido en el tiempo cero fue del 98 al 102 %, en base seca. La desviación estándar relativa de las recuperaciones en cada tiempo no superó el 2 % (cuadro 3), lo que es equivalente a un buen resultado.

**Cuadro:** 3. Resumen de los resultados de estabilidad en la solución estándar de valoración, la solución muestra de valoración y la solución estándar de impurezas orgánicas del fluconazol

Estándar de valoración				
Criterio de aceptación	Condiciones	Correlación (%)	Correlación (%)	Recobro (%)
		0 horas	24 horas	24 horas
La correlación debe estar entre 98,0 y 102,0 %.	Ambiente	100,5	100,0	99,5
El recobro calculado entre la correlación promedio de la frecuencia evaluada y la correlación promedio inicial debe estar entre 98,0 y 102,0 %.	Refrigeración		100,1	99,6
Solución muestra				
Criterio de aceptación	Condiciones	Contenido inicial Contenido promedio Recobro (% ± RSD)		
		(% ± RSD)	(% ± RSD) 24 horas	24 horas
El RSD de las muestras en cada tiempo no es mayor de 2,0 %.	Ambiente	101,5 ± 0,1	100,9 ± 0,2	99,4
El recobro calculado entre el contenido promedio de la frecuencia evaluada y el contenido promedio inicial debe estar entre 98,0 y 102,0 %.	Refrigerado		100,6 ± 0,1	99,1
Estándar de impurezas orgánicas				
Criterio de aceptación	Condiciones Compuesto	Área	Área	Recobro (%)
		0 horas	24 horas	24 horas
El recobro calculado entre la correlación promedio de la frecuencia evaluada y la correlación promedio inicial debe estar entre 98,0 y 102,0 %.	Ambiente	fluconazol C.R. A fluconazol C.R. B fluconazol C.R. C fluconazol	7,618685 0,590195 12,547291 0,652845	7,632293 0,580262 12,540827 0,647447
	Refrigeración	fluconazol C.R. A fluconazol C.R. B fluconazol C.R. C fluconazol	7,618685 0,590195 12,547291 0,652845	7,624229 0,587135 12,506710 0,656819

RSD: desviación estándar relativa; C.R.: compuesto relacionado

### Validación de la metodología

**Figura 3:**
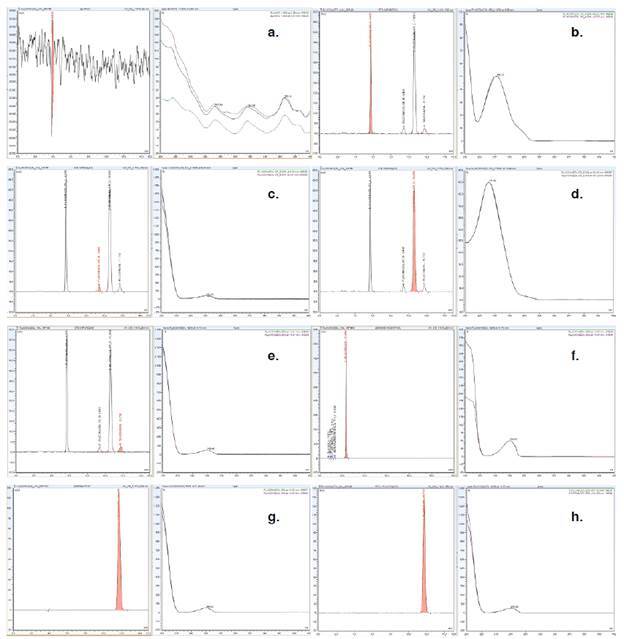
Perfiles cromatográficos de: **(a)** solución usada como blanco, **(b)** estándar de impurezas de fluconazol - compuesto relacionado A, **(c)** estándar de impurezas de fluconazol - compuesto relacionado B, **(d)** estándar de impurezas de fluconazol - compuesto relacionado C, **(e)** estándar de impurezas fluconazol, **(f)** muestra de impurezas de fluconazol, **(g)** estándar de valoración de fluconazol, **(h)** muestra de valoración de fluconazol

*Especificidad.* Se determinó la metodología analítica empleada para cuantificar impurezas orgánicas presentes en el fluconazol materia prima; no se evidenciaron señales que pudieran interferir en la identificación y cuantificación del analito de interés (figura 3, a-h). La resolución no fue menor de 1,5, lo que indica un buen resultado. La pureza espectral del analito de interés se demostró con un *factor match* cercano a 1.000 para las pruebas realizadas en el *software Chromeleon.*

*Linealidad del sistema para la valoración y detección de impurezas orgánicas de fluconazol.* El coeficiente de correlación fue mayor de 0,999 para la valoración, y mayor o igual a 0,997 para las impurezas orgánicas. Se confirmó la ausencia de error sistemático, ya que la correlación entre el límite inferior del rango (0,45 mg/ml), la concentración nominal (0,5 mg/ml) y el límite superior del rango (0,55 mg/ml) frente al estándar 1, fue de 98-102 % para la valoración y fue del 80 al 120 % para las impurezas orgánicas. Los resultados de la pendiente de la regresión lineal, el coeficiente de correlación, el gráfico de la suma residual de los cuadrados y la dispersión aleatoria alrededor de la línea del cero, que fue de ± 2 % para la valoración y de ± 10 % para las impurezas orgánicas, confirmaron la linealidad del sistema (cuadro 4).

**Cuadro 4:** Resumen de los resultados de la linealidad del sistema en la valoración del fluconazol y la detección de sus impurezas orgánicas.

Criterio de aceptación	Valoración			Impurezas orgánicas			
				fluconazol			
				C.R. A	C.R. B	C.R. C	API
	Nivel	Resultado	Nivel		Resultado		
	(%)	(%)	(%)		(%)		
Pendiente		73,601		766,69	62,028	1262,3	69,31!
r ≥ 0,999		0,99957		0,99991	0,99800	0,99995	0,99712
Evaluación del intercepto (ausencia de errores sistemáticos):	90	100,1	LQ	104,7	105,1	106,0	102,2
		100,5		105,6	104,2	109,8	100,9
La correlación entre el estándar y cada una de las inyecciones de los niveles evaluados, debe estar entre 98,0 y 102,0 % para la valoración, y entre 80,0 y 120,0 % para las impurezas orgánicas del fluconazol.		100,1		105,0	104,6	107,4	101,8
	100	100,2	100	102,6	102,4	105,7	98,1
		100,1		102,7	102,5	105,6	98,4
		100,1		103,0	100,5	105,6	100,0
	110	100,3	120	101,3	104,9	104,8	102,9
		100,2		101,5	103,2	105,4	102,8
		100,1		101,6	104,3	105,5	103,2
La dispersión aleatoria alrededor de la línea del cero ± 2,0 % para la valoración y ± 10,0 % para las impurezas orgánicas del fluconazol.	90	-0,3	LQ	-8,3	0,4	-3,0	1,5
		0,1		-7,5	-0,4	0,4	0,1
		0,1		-8,0	0,0	-1,7	1,0
	96	-0,1	90	0,6	2,1	-0,2	0,8
		0,0		0,2	0,9	0,1	-0,1
		0,4		0,8	1,0	0,2	1,6
	100	0,0	100	0,3	-1,5	0,0	-3,8
		-0,1		0,4	-1,5	-0,1	-3,5
		-0,1		0,7	-3,4	0,0	-2,0
	106	0,0	110	0,7	-0,7	1,1	0,9
		-0,5		-0,1	2,1	0,1	1,2
		0,1		-0,2	0,5	0,1	0,9
	110	0,2	120	-0,9	1,0	-0,8	0,6
		0,1		-0,8	-0,7	-0,2	0,5
		0,0		-0,6	0,4	-0,1	0,9

API: ingrediente farmacéutico activo; LQ: límite de cuantificación

*Exactitud para la valoración de fluconazol y sus impurezas orgánicas.* El porcentaje de recuperación individual evaluado versus la calibración externa en la valoración para el fluconazol fue del 97 al 103 %, y para la cuantificación de las impurezas orgánicas, fue del 80 al 120 %. El porcentaje de recuperación media de cada nivel y el global versus el de la calibración externa fue del 98 al 102 % para la valoración del fluconazol, y del 80 al 120 % en la cuantificación de las impurezas orgánicas.

La desviación estándar relativa de las determinaciones de cada nivel, no fue mayor del 2 % para la valoración, mientras que, para las impurezas orgánicas, se estableció de acuerdo con la relación entre la concentración límite de impurezas y el límite de cuantificación: si la concentración límite de impurezas estaba entre 1 y 2 veces el límite de cuantificación, la desviación estándar relativa era menor o igual a 25 %; entre 2 y 10 veces el límite de cuantificación, la desviación estándar relativa era menor o igual a 15 %; y entre 10 y 20 veces el límite de cuantificación, la desviación estándar relativa era menor o igual a 5 %.

*Precisión para valorar y detectar impurezas orgánicas de fluconazol (repetibilidad del sistema, del método y precisión intermedia de fluconazol).* La desviación estándar relativa de los resultados obtenidos de las seis réplicas realizadas no fue mayor del 2 % para la valoración y del 5 % para las impurezas orgánicas en la repetibilidad del sistema. En la valoración no se obtuvo una desviación estándar relativa mayor de 2 % entre los datos individuales por serie para la repetibilidad del método. Tampoco fue mayor del 3 % para la precisión intermedia (comparación entre el analista 1 y el analista 2). La diferencia absoluta entre las medias de los dos analistas no fue mayor del 2 % para la prueba de valoración (cuadro 5).

**Cuadro 5:** Resumen de los resultados de la precisión del sistema en la valoración del fluconazol y la detección de sus impurezas orgánicas

Criterio de aceptación	Valoración	Impurezas orgánicas								
		Fluconazol								
			C.R. A		C.R. B		C.R. C		API	
				Resultado (%)						
RSD de la reproducibilidad del sistema ≤ 2,0 % para la valoración y ≤ 5,0 % para las impurezas orgánicas del fluconazol	A1S1	A1S2	A1S1	A1S2	A1S1	A1S2	A1S1	A1S2	A1S1	A1S2
	0,2	0,2	0,3	1,1	3,0	3,3	0,3	0,7	3,0	3,5
	A2S1	A2S2	A2S1	A2S2	A2S1	A2S2	A2S1	A2S2	A2S1	A2S2
	1,9	0,1	0,2	0,6	3,2	4,9	0,6	0,9	4,2	4,8
RSD de la reproducibilidad del método ≤ 2,0 % para la valoración y ≤ 5,0 % para las impurezas orgánicas del fluconazol (para el fluconazol y el fluconazol C.R. B ≤ 25,0 %)	A1S1	A1S2	A1S1	A1S2	A1S1	A1S2	A1S1	A1S2	A1S1	A1S2
	0,3	0,4	0,4	0,8	1,4	1,3	0,5	1,0	1,2	0,8
	A2S1	A2S2	A2S1	A2S2	A2S1	A2S2	A2S1	A2S2	A2S1	A2S2
	0,5	0,5	0,4	0,9	0,9	1,3	4,3	1,0	1,7	1,0
	Promedio A1									
La diferencia absoluta entre las medias de los dos analistas debe ser ≤ 2,0 % para la valoración y ≤ 10,0 % para las impurezas orgánicas del fluconazol.	99,7		101,3		100,3		99,9		99,5	
	Promedio A2									
	100,9		100,6		102,1		101,5		98,2	
	Diferencia absoluta									
	1,2	0,7		1,8		1,6			1,3	

RSD: desviación estándar relativa; A1: analista 1; A2: analista 2; S1: serie 1; S2: serie 2

El coeficiente de variación entre los datos individuales por serie para la repetibilidad del método en las impurezas orgánicas, no fue mayor al establecido de acuerdo con la relación mencionada entre la concentración límite de impurezas y el límite de cuantificación: si la concentración límite de impurezas estaba entre 1 y 2 veces el límite de cuantificación, la desviación estándar relativa era menor o igual al 25 %; entre 2 y 10 veces el límite de cuantificación, la desviación estándar relativa era menor o igual al 15 %; entre 10 y 20 veces el límite de cuantificación, la desviación estándar relativa era menor o igual al 10 %; y si la concentración límite de impurezas superaba más de 20 veces el límite de cuantificación, la desviación estándar relativa era menor o igual al 5 %.

La precisión intermedia no fue mayor del 3 % (comparación entre el analista 1 y el analista 2). El coeficiente de variación entre series (precisión intermedia) no fue mayor del establecido según la relación entre la concentración límite de impurezas y el límite de cuantificación: si la concentración límite de impurezas estaba entre 1 y 2 veces el límite de cuantificación, la desviación estándar relativa era menor o igual al 30 %; entre 2 y 10 veces el límite de cuantificación, la desviación estándar relativa era menor o igual al 20 %; entre 10 y 20 veces el límite de cuantificación, la desviación estándar relativa era menor o igual al 15 %; y si la concentración límite de impurezas superaba más de 20 veces el límite de cuantificación, la desviación estándar relativa era menor o igual al 10 %. La diferencia absoluta entre las medias de los dos analistas no fue mayor del 10 % para la prueba de impurezas orgánicas (cuadro 5).

*Solidez (robustness).* La precisión intermedia formalmente conocida como solidez *(robustness),* estuvo dentro de los parámetros establecidos para las pruebas de valoración e impurezas orgánicas del fluconazol (cuadro 5). Estos parámetros, según indicaciones de la Conferencia Internacional de Armonización, se tomaron en el mismo laboratorio, pero en días, con analistas y con equipos diferentes ^14^, en búsqueda de la coincidencia de los resultados.

## Discusión

Las soluciones presentaron estabilidad a temperatura ambiente y de refrigeración a las 24 horas, cumpliendo los parámetros de idoneidad. En la estandarización de la metodología, se obtuvo gran eficiencia del filtro hidrofílico de PVDF de 0,45 µm, ya que este no presentó retención del analito, ni tampoco generó señales adicionales que pudieran interferir al momento de cuantificar e identificar los analitos.

De acuerdo con los resultados obtenidos en las pruebas de degradación forzada, se confirmó que el método es específico y selectivo, y por lo tanto, permite la identificación inequívoca de los analitos de interés. Todos los resultados de las degradaciones se encontraron dentro del límite permitido. Además, se confirmó la naturaleza química estable del analito sometido a condiciones de estrés por hidrólisis ácida, hidrólisis básica, oxidación, termólisis y fotólisis, de manera que este parámetro también indica estabilidad de las soluciones.

La pureza espectral del analito de interés se demostró en todas las muestras sometidas a degradación forzada, ya que se obtuvieron valores de *factor match (software Chromeleon)* cercanos a 1.000. Asimismo, los reactivos usados para la prueba no interfirieron al momento de emplear el método.

La gran estabilidad de la molécula de fluconazol puede deberse a sus propiedades fisicoquímicas: tiene un peso molecular de 306,27 g/mol, una carga formal de cero, un área de superficie polar de 81,6 Å^2^ que facilita la permeación de la molécula en las membranas celulares y que actúe sobre los receptores del sistema nervioso central ^22^; un punto de ebullición de 579,8 °C, un punto de fusión entre 138 y 140 °C y un pKa igual a 1,76 ^1^.

La prueba de estabilidad demostró que la solución estándar de valoración, la solución muestra de valoración y la solución estándar de impurezas para los compuestos relacionados A, B y C, y fluconazol (materia prima), son estables a temperatura ambiente y en refrigeración (2-8 °C) durante 24 horas. La recuperación de las soluciones no se vio afectada a las 24 horas, ni el contenido inicial (manteniendo el contenido promedio), al igual que la desviación estándar relativa entre los diferentes contenidos de las muestras (cuadro 3).

En la validación de la metodología, se demostró la especificidad del método (figura 3, a-h). Ningún pico del diluyente interfirió en la identificación y la cuantificación de los compuestos relacionados A, B y C, y el fluconazol (materia prima). El método es selectivo y los reactivos usados para la prueba no obstaculizan su aplicación. Se demostró la linealidad del sistema para fluconazol en las pruebas de valoración y para los compuestos relacionados A, B y C, y fluconazol (materia prima) en las impurezas orgánicas. Los gráficos de los residuos mostraron una dispersión aleatoria alrededor de la línea cero para la valoración del ingrediente farmacéutico activo y las impurezas orgánicas del fluconazol no mostraron un patrón. La evaluación del intercepto y la ausencia de error sistemático presentaron una correlación entre el estándar y cada una de las inyecciones de los niveles evaluados para la valoración del ingrediente farmacéutico activo y las impurezas orgánicas del fluconazol (cuadro 4).

El método es exacto para el fin propuesto porque los porcentajes de recuperación de la prueba de valoración están dentro del rango establecido, así como aquellos de los compuestos relacionados A, B y C, y el fluconazol (materia prima) obtenidos en la prueba de impurezas orgánicas. La desviación estándar relativa entre los contenidos de las determinaciones de cada nivel fue menor del 2 %, tanto para la valoración como para las impurezas orgánicas.

Se demostró la precisión de las pruebas a partir de la repetibilidad y la reproducibilidad del método para la cuantificación del fluconazol -y los compuestos relacionados- en la valoración y en las impurezas orgánicas. La metodología evaluada fue precisa ya que la repetibilidad del sistema no superó una desviación estándar relativa del 2 % en la valoración y del 5 %, en las impurezas orgánicas. En la repetibilidad del método, se obtuvo una desviación estándar relativa inferior al 2 % en la valoración e inferior al 5 % para el compuesto relacionado A y C, mientras que, para el compuesto relacionado B y el fluconazol (materia prima), la repetibilidad del método tuvo una desviación estándar relativa inferior al 25,0 %. En la precisión intermedia, se obtuvo una desviación estándar relativa menor del 3 % en la valoración; y menor del 10 % para los compuestos relacionados A y C, e inferior al 30 % para el fluconazol compuesto relacionado B y el fluconazol (materia prima), en la detección de impurezas.

La diferencia absoluta entre las medias de los dos analistas fue menor del 2 % en la valoración y menor del 10 % en las impurezas orgánicas (cuadro 5). La metodología usada es lo suficientemente robusta al no verse afectada por variaciones pequeñas, aunque deliberadas, lo que indica su aptitud en condiciones normales de uso.

En este trabajo se desarrolló el método de análisis por HPLC-DAD de la materia prima fluconazol (ingrediente farmacéutico activo). Se estableció un tiempo total de la corrida cromatográfica por inyección de 15 minutos, que es un tiempo de respuesta rápido anclado a una mayor eficiencia de los equipos y un impacto ambiental más bajo al generar menos desechos de reactivos contaminantes. Se establecieron los rangos probables de tiempo de retención para cada uno de los picos de interés del fármaco, así: fluconazol compuesto relacionado A: 5,4 a 5,8 minutos; fluconazol compuesto relacionado B: 8,8 a 9,7 minutos; fluconazol compuesto relacionado C: 9,6 a 10,9 minutos; y fluconazol (materia prima): 10,8 a 12,4 minutos.

En la prueba de valoración, fue posible cuantificar fluconazol con precisión en un rango de 0,45 a 0,55 mg/ml, respecto a la concentración analítica de trabajo (0,50 mg/ml), con una exactitud de 100 % ± 2,0 % y una precisión menor o igual al 2 % del valor notificado. En la detección de impurezas orgánicas, se realizó la cuantificación precisa del fluconazol en un rango de 5,85 a 12,0 µg/ml; para el compuesto relacionado A, en un rango de 0,25 a 12,0 µg/ml; para el B, en un rango de 5,50 a 12,0 µg/ml; y para el C, en un rango de 0,30 a 12,0 µg/ml, respecto a la concentración analítica de trabajo (10,0 µg/ml), con una exactitud del 100 % ± 20 % y una precisión menor del 10 % del valor notificado.

Se validó la metodología por HPLC-DAD en fase inversa para la valoración, la identificación y la cuantificación de las impurezas orgánicas especificas e inespecíficas del fluconazol en materia prima. Asimismo, se proporcionaron los parámetros por evaluar y los criterios de aceptación, con los cuales se demostró que el método proporciona resultados analíticos veraces en condiciones de uso reales en un clima tropical, y valores exactos y reproducibles dentro del rango determinado. Además, se probó que el método validado es lo suficientemente selectivo y estable en las variaciones propuestas.
